# Prevalence and Associated Factors of Insulin Resistance in Adults from Maracaibo City, Venezuela

**DOI:** 10.1155/2016/9405105

**Published:** 2016-08-04

**Authors:** Valmore Bermudez, Juan Salazar, María Sofía Martínez, Mervin Chávez-Castillo, Luis Carlos Olivar, María José Calvo, Jim Palmar, Jordan Bautista, Eduardo Ramos, Mayela Cabrera, Freddy Pachano, Joselyn Rojas

**Affiliations:** ^1^Endocrine and Metabolic Diseases Research Center, School of Medicine, University of Zulia, Maracaibo 4004, Venezuela; ^2^Morphologic Sciences Department and Pediatric Surgery Department, School of Medicine, University of Zulia, Maracaibo 4004, Venezuela

## Abstract

*Background and Aim*. Insulin resistance (IR) is a prominent pathophysiologic component in a myriad of metabolic disorders, including obesity, prediabetes, and type 2 diabetes mellitus, which are common in our locality. The objective of this study was to determine the prevalence of IR and factors associated with this condition in an adult population from Maracaibo city, Venezuela.* Methodology*. A cross-sectional, descriptive study with multistaged randomized sampling was carried out in 2026 adults. IR was defined as HOMA2-IR ≥ 2. A multiple logistic regression model was constructed in order to evaluate factors associated with IR.* Results*. The prevalence of IR was 46.5% (*n* = 943), with 46.7% (*n* = 450) in the general population, 46.4% (*n* = 493) in females, and 47.90% (*n* = 970) in males (*p* = 0.895). IR prevalence tended to increase with age and was significantly greater in subjects aged ≥30 years (*χ* ^2^ = 16.726; *p* = 2.33 × 10^−4^). Employment, alcohol consumption, obesity, high triacylglycerides, low HDL-C, and dysglycemia were associated with greater odds of IR, whereas a high level of physical activity appeared to be weak protective factor against IR.* Conclusions*. The prevalence of IR is elevated in our locality. The main determinants of this condition appear to be the presence of obesity, high triacylglycerides, low HDL-C, dysglycemia, and alcohol intake.

## 1. Introduction

The term* insulin resistance* (IR) was coined in 1936 to describe a metabolic disturbance characterized by decreased cellular responsiveness to insulin signaling in insulin-dependent tissues such as skeletal muscle, liver, and adipose tissue [1]. IR has now been established to play a key role in pancreatic *β* cell dysfunction and type 2 diabetes mellitus (DM2) [2]. This disease represents one of the leading causes of mortality in the adult population worldwide [3], with alarming projections for the future, with an estimated prevalence of 33% in adults in the USA by 2050 [4]. Furthermore, IR is a fundamental pathophysiologic component of several other endocrine-metabolic disorders, such as hypertension, dyslipidemias, polycystic ovary syndrome, metabolic syndrome, and cardiovascular disease [5, 6].

In light of the great morbidity and mortality rates associated with these disorders, prominent scientific effort has been directed to the refinement of tools for the detection and assessment of IR. In this regard, HOMA2-IR [7] stands as one of the most broadly accepted models in research settings yet remains relatively underutilized in our local clinical scenario. In this context, previous analyses by our research group have estimated specific cutoffs for this variable in our population [8], laying the groundwork for their potential clinical and epidemiologic use.

IR is intimately linked with psychobiologic habits such as smoking, alcohol consumption, hypercaloric diets, and physical inactivity. In turn, these factors can be strongly conditioned by demographic, social, and economic aspects, which shape daily human living. Thus, the objective of this study was to determine the prevalence and determinants of IR in the adult population of Maracaibo city, Venezuela.

## 2. Materials and Methods

### 2.1. Sample Selection

This report is part of the Maracaibo City Metabolic Syndrome Prevalence Study, a cross-sectional study whose purpose is to identify MS and cardiovascular risk factors in the adult population of Maracaibo, the second largest city of Venezuela. The sample (1,986 individuals) was calculated based on estimations of the city's population by our National Institute of Statistics (1,428,043 inhabitants for the year 2007). A total of 244 subjects (12%) were added for oversampling, in order to increase accuracy of the estimates obtained from smaller subgroups from the overall sample, amounting to a total of 2,230 individuals. Maracaibo city is divided into parishes, each of which was proportionally sampled with a multistaged cluster method: In the first stage, clusters were represented by sectors from each of the 18 parishes, selecting 4 from each parish by simple randomized sampling. In the second phase, clusters were represented by city blocks within each sector, which were selected by simple randomized sampling using a random number generation tool. Further details of the sampling process have been previously published elsewhere [9].

### 2.2. Ethical Considerations

All participants signed written consent prior to undergoing physical examination and blood sample collection. All procedures were approved by the Ethics Committee of the Endocrine and Metabolic Diseases Research Center of The University of Zulia, Maracaibo, Venezuela.

### 2.3. Subject Evaluation

Data were collected through completion of a full clinical record carried out by trained personnel, which encompassed interrogation regarding ethnic origin, marital status (single, married, cohabiting, or divorced), occupational status (employed or unemployed), and educational status, under the following definitions: (a) illiterate, subjects with no reading or writing skills; (b) primary education, those who attended or finished primary or middle school; (c) secondary education, those who attended or finished high school; and (d) high education, those who attended or finished college/university [9]. Socioeconomic status was assessed with the Graffar scale modified by Méndez-Castellano for the Venezuelan population [10]. This tool allows stratification of the population according to four variables: occupation of the head of household, educational level of the subject's parents, income source, and housing conditions. Each of these items is rated in a Likert-type scale from 1 to 5 (1 representing the best possible scenarios and 5 the worst). The scores for each item are tallied into a total integrated score ranging from 4 to 20 points, categorized as follows: Stratum I or High Class (4–6 points); Stratum II or Middle-High Class (7–9 points); Stratum III or Middle Class (10–12 points); Stratum IV or Relative Poverty (13–16 points), and Stratum V or Critical Poverty (17–20 points).

#### 2.3.1. Smoking Habit

Based on information obtained during the clinical interview, subjects were categorized by their smoking habits as follows [11]: (a) nonsmokers, individuals who had never smoked or had smoked <100 cigarettes in their lifetime; (b) current smokers, subjects who had smoked ≥100 cigarettes in their lifetime or reported current habitual smoking at the time of evaluation or had quit smoking less than one year prior to our assessment; and (c) past smokers, individuals who had consumed ≥100 cigarettes in their lifetime and quit over one year prior to our questioning.

#### 2.3.2. Alcohol Consumption

For the assessment of alcohol intake, subjects were asked to estimate the amount of alcoholic drinks they consumed within a month, with the approximate quantity and frequency of daily intake for each type of drink: beer, spirits, and wine and its derivatives. Accounting for the standard content of alcohol grams in each kind of beverage [12], daily intake of alcohol grams was calculated through the following formula [13]:

(1) $$\begin{array}{c} \frac{\text{Daily consumed (mL)}\times \text{degrees of alcohol}\times 0.8}{100}, \end{array}$$

where 0.8 is a constant representing ethanol density in drinks. Based on this estimation, drinkers were defined as subjects who consumed ≥1 gram of alcohol daily [14].

#### 2.3.3. Physical Activity

Physical activity (PA) was assessed with the International Physical Activity Questionnaire (IPAQ) [15]. For statistical analysis, PA was evaluated in 4 domains: occupational, household, transport, and leisure. In each of these domains, subjects were categorized as follows: (a) inactive, MET/week = 0, or (b) active, MET/week >0. The latter were then subcategorized by gender-specific MET/week quintiles in each domain (Table 1).

#### 2.3.4. Blood Pressure

Blood pressure (BP) was taken with subjects sitting down with their feet on the floor following 15 minutes of rest, determined through the auscultatory method with a calibrated mercury sphygmomanometer, identifying Korotkoff's phases I and V as systolic and diastolic BP, respectively. BP was determined 3 times, with 15 minutes in between each take, on two different days; results were classified by the Eighth Joint National Committee on Prevention, Detection, Evaluation, and Treatment of High Blood Pressure (JNC-8) guidelines [16].

#### 2.3.5. Anthropometry

An electrical bioelectric scale was used to obtain weight (Tanita, TBF-310 GS Body Composition Analyzer, Tokyo, Japan). Height was measured using a calibrated metric measurement tape, with the subject standing up barefoot. Body Mass Index (BMI) was calculated with the following formula: [weight/height^2^] expressing results as kg/m^2^. According to their BMI, subjects were sorted in 3 categories: (a) BMI ≤ 24.9; (b) 25–29.9; and (c) ≥30 [17]. Waist circumference (WC) was evaluated with calibrated measuring tapes in accordance with the anatomical landmarks proposed by the USA National Institutes of Health protocol [18].

#### 2.3.6. Laboratory Analysis

Overnight fasting determination of glucose, total cholesterol, triacylglycerides (TAG), and HDL-C was done with an automated analyzer (Human Gesellschaft für Biochemica und Diagnostica mbH, Germany); the intra-assay variation coefficients for total cholesterol, TAG, and HDL-C were 3%, 5%, and 5%, respectively. LDL-C and VLDL-C levels were calculated applying Friedewald's formula [19] but when TAG levels were <400 mg/dL. LDL-C concentrations were directly measured through lipoprotein electrophoresis and densitometry with a BioRad GS-800 optical densitometer. Insulin was quantified using ultrasensitive ELISA double-sandwich methodology (DRG Instruments GmbH, Germany, Inc.).

#### 2.3.7. Diagnosis of Metabolic Syndrome

The metabolic syndrome was defined by the criteria from the IDF/NHLBI/AHA/WHF/IAS/IASO-2009 consensus [20], which require the presence of ≥3 of the following components: (a) low HDL-C, <50 mg/dL in females or <40 mg/dL in males; (b) high TAG, ≥150 mg/dL; (c) elevated WC, ≥80 cm in females or ≥90 cm in males; (d) hyperglycemia, fasting glycemia ≥100 mg/dL or personal history of type 2 diabetes mellitus or prescription of hypoglycemic drugs; and (e) High Blood Pressure, BP ≥130/85 mm/Hg or previously diagnosed hypertension or prescription of antihypertensive drugs.

#### 2.3.8. Assessment of Insulin Resistance

HOMA2-IR was utilized for the evaluation of IR as proposed by Levy et al. [21], computed with the HOMA-Calculator version 2.2.2 software application. IR was defined as HOMA2-IR ≥ 2 [8].

### 2.4. Statistical Analysis

Qualitative variables were expressed as absolute and relative frequencies, evaluating association through Pearson's Chi-squared (*χ* ^2^) test, while the *Z* test for proportions was used to assess differences between proportions. Quantitative variables were evaluated for distribution normality with Geary's test and were expressed as arithmetic means ± SD. Variables with nonnormal distribution underwent logarithmic transformation; when normalization could not be achieved, these variables were expressed as medians (25th percentile–75th percentile). Student's *t*-test or Mann-Whitney's *U* tests were applied to evaluate differences between means or medians, respectively.

A multiple logistic regression model was constructed in order to estimate odds ratios (confidence interval 95%) for the presence of IR, adjusted by gender, age groups, ethnic groups, socioeconomic status, educational status, marital status, occupational status, smoking habits, leisure-domain PA, glycemic status, presence of high TAG, presence of low HDL-C, JNC-8 classification, presence of elevated WC, and BMI classification. Data were analyzed with the Statistical Package for the Social Sciences (SPSS) version 21 for Windows (IBM Inc., Chicago, IL), and the results were considered statistically significant when *p* < 0.05.

## 3. Results

### 3.1. Characteristics of the General Population

The sociodemographic and psychobiologic features of the sample are summarized in Table 2, while metabolic and anthropometric aspects are presented in Table 3. A total of 2026 individuals were studied: 52.10% (*n* = 1056) females and 47.90% (*n* = 970) males. The mean age was 39.69 ± 15.37 years (41.06 ± 15.68 years for women, 38.20 ± 14.89 years for men).

### 3.2. HOMA2-IR by Gender and Age Groups

**Figure 1:**
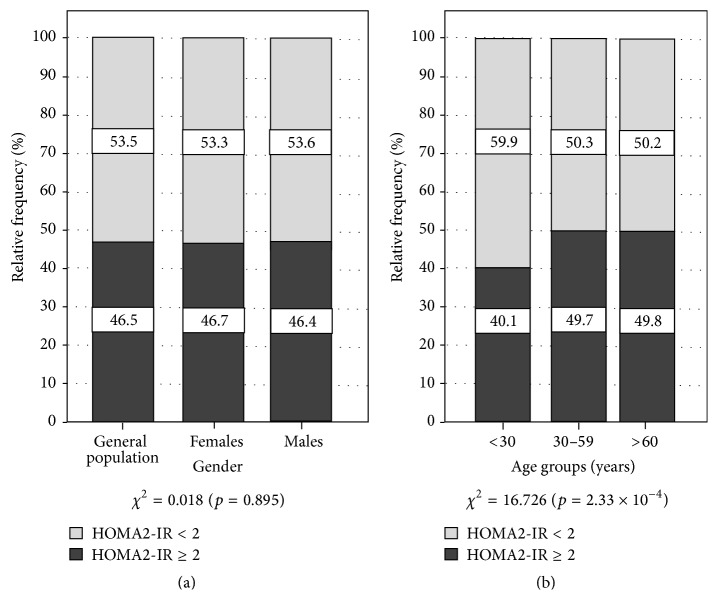
Prevalence of insulin resistance by gender and age groups. Maracaibo, Venezuela. 2014. *Z* Test for proportions: gender: nonsignificant. Age groups: <30 years (HOMA2-IR < 2 versus HOMA2-IR ≥ 2; *p* < 0.05); 30–59 years (HOMA2-IR ≥ 2 versus HOMA2-IR < 2; *p* < 0.05).

The prevalence of IR was 46.5% (*n* = 943) in the general population, with 46.7% (*n* = 493) in women and 46.4% (*n* = 450) in men (*χ* ^2^ = 0.018; *p* = 0.895) (Figure 1(a)). On the other hand, Figure 1(b) illustrates the prevalence of IR by age groups: A significantly greater proportion of IR was found in the groups aged 30–59 years (49.7%, *n* = 559) and ≥60 years (49.8%, *n* = 114), in comparison to individuals aged <30 years (40.1%, *n* = 270) (*χ* ^2^ = 16.726; *p* = 2.33 × 10^−4^).

### 3.3. HOMA2-IR and Other Sociodemographic Factors

When assessing the epidemiologic behavior of IR according to sociodemographic factors (Table 4), an association was found only in regard to marital status (*χ* ^2^ = 17.293, *p* = 0.001), with a significantly greater proportion of IR subjects among married individuals (51.5% versus 48.5% individuals with HOMA2-IR < 2).

### 3.4. HOMA2-IR and Psychobiologic Habits

**Figure 2:**
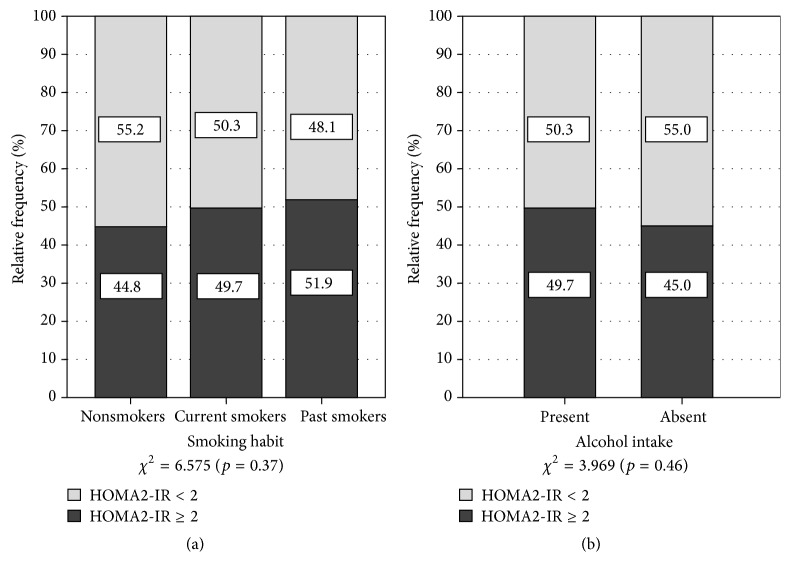
Prevalence of insulin resistance in the general population by smoking habits and alcohol intake. Maracaibo, Venezuela. 2014. *χ* ^2^ = Pearson's Chi-squared test. *Z* test for proportions: smoking habits: nonsmokers (HOMA2-IR < 2 versus HOMA2-IR ≥ 2; *p* < 0.05); past smokers (HOMA2-IR ≥ 2 versus HOMA2-IR < 2; *p* < 0.05). Alcohol intake: present (HOMA2-IR ≥ 2 versus HOMA2-IR < 2; *p* < 0.05); absent (HOMA2-IR < 2 versus HOMA2-IR ≥ 2; *p* < 0.05).

Regarding smoking habits (Figure 2(a)), no link was found with the presence of IR (*χ* ^2^ = 6.575; *p* = 0.37). Nevertheless, we observed a greater proportion of IR subjects among past smokers (51.9% versus 48.1% without IR; *p* < 0.05). Conversely, the absence of IR was prevalent among nonsmokers (55.2% versus 44.8% with IR; *p* < 0.05). With respect to alcohol intake and IR (Figure 2(b)), no statistically significant association was found (*χ* ^2^ = 3.969; *p* = 0.46). Similarly, no link was ascertained between IR and PA in the home (*χ* ^2^ = 2.27; *p* = 0.81), work (*χ* ^2^ = 1.82; *p* = 0.87), and transport (*χ* ^2^ = 8.60; *p* = 0.12) domains (Table 5). However, a significant association was ascertained between the presence of IR and leisure-domain PA (*χ* ^2^ = 23.24;  *p* = 0.003), with a higher proportion of non-IR individuals in the high and very high PA categories in this domain (61.3%, *n* = 92 and 65.5%, *n* = 110, resp., versus IR subjects; *p* < 0.05).

### 3.5. HOMA2-IR and Metabolic Characteristics

The presence of HOMA2-IR ≥ 2 was found to be associated with various metabolic characteristics (Table 6), being significantly more prevalent among subjects with BMI ≥ 30 kg/m^2^ (68.8% versus 31.2% in subjects without IR; *p* < 0.05), high TAG (62.8% versus 37.2%; *p* < 0.05), low HDL-C (52.5% versus 47.5%; *p* < 0.05), elevated WC (67.7% versus 36.3%; *p* < 0.05), hypertension (57.9% versus 42.1%; *p* < 0.05), impaired fasting glucose (37.6% versus 62.4%; *p* < 0.05), and DM2 (78.7% versus 21.3%; *p* < 0.05).

### 3.6. HOMA2-IR and Biologic and Anthropometric Variables

Table 7 displays the behavior of several biologic and anthropometric characteristics by IR status. Individuals with this disorder had significantly higher age (*p* = 3.15 × 10^−5^), BMI (*p* = 13 × 10^−5^), WC (*p* = 4.49 × 10^−54^), fasting blood glucose (*p* = 3.41 × 10^−39^), systolic BP (*p* = 2.33 × 10^−8^), diastolic BP (*p* = 2.93 × 10^−11^), and hs-CRP (*p* = 4.51 × 10^−7^) than the non-IR population. A similar pattern was found regarding serum lipids, especially TAG (154.48 ± 122.02 versus 110.70 ± 76.27; IR and non-IR subjects, *p* = 5.45 × 10^−29^) and HDL-C, which was significantly lower in IR individuals (41.97 ± 10.95 versus 45.90 ± 12.58; *p* = 3.67 × 10^−14^).

### 3.7. Risk Factors for Insulin Resistance in Maracaibo City

The risk factors for the presence of IR in our population are shown in Table 8. Following adjustment of the multivariate model, the age groups of 30–59 years (OR: 0.65, CI 95%: 0.50–0.85; *p* < 0.01) and ≥60 years (OR: 0.42, CI 95%: 0.28–0.65; *p* < 0.01) were associated with lower odds of IR. On the other hand, the female gender (OR: 1.29, CI 95%: 1.02–1.65; *p* = 0.04) and employed occupational status (OR: 1.45, CI 95%: 1.15–1.83; *p* < 0.01) were linked with higher risk of IR. The sole psychobiologic factor independently related to IR was alcohol intake (OR: 1.41, CI 95%: 1.10–1.79; *p* < 0.01). Similarly, high TAG (OR: 1.77, CI 95%: 1.38–2.27; *p* < 0.01), low HDL-C (OR: 1.31, CI 95%: 1.06–1.64; *p* = 0.01), and BMI ≥30 kg/m^2^ (OR: 4.33, CI 95%: 2.97–6.29; *p* < 0.01), as well as impaired fasting glucose and DM2, were also associated with increased odds of IR in our population. Furthermore, no relevant differences were revealed after adjustment of a second model with exclusion of diabetic subjects.

## 4. Discussion

In recent years, a myriad of biologic, psychological, sociodemographic, and economic factors have driven the* epidemiologic transition*, which features a shift in the major worldwide causes of mortality from infectious diseases to chronic-degenerative disorders [22]. This group includes cardiovascular disease and DM2, where IR plays a fundamental role in their pathophysiology, in close association with Westernized lifestyles such as physical inactivity and hypercaloric diets [1]. Thus, detection and management of this condition have become a priority in primary care. Nevertheless, its epidemiologic behavior remains incompletely elucidated in many populations, including our locality.

We found the prevalence of IR in Maracaibo to be relatively elevated, in harmony with other reports within our country, 54.8% in Valencia city [23], 50.6% in a community from Tinaquillo [24], and up to 61.2% in a Canarian-Venezuelan population [25]. This may be explained by the growing prevalence of obesity and other cardiovascular risk factors in Venezuela [26], as well as methodological limitations and heterogeneity.

Moreover, IR prevalence is widely variable across demographics: whereas European populations appear to boast lower prevalence of IR, estimated at 17% in a Danish study, for example, [27], it has been reported to be as high as 51% in Iran, West Asia [28]. On the other hand, IR prevalence has been estimated at 39.1% in Hispanic subjects residing in Texas, USA [29]. Indeed, differing genetic, epigenetic, and sociocultural factors are important determinants of insulin sensitivity [30, 31]. In addition, utilization of IR-assessing methods different from HOMA2-IR or arbitrary cutoffs may also contribute to these disparities [32].

In our sample, IR prevalence was similar between genders, diverging from findings in a Danish population [27] and the* Framingham Heart Study* [33], where men showed a higher prevalence, ostensibly attributed to the protective role of estrogens in the development of IR [34]. However, in our study, multivariate analysis revealed women to be at greater risk of IR, echoing a previous report by Rojas et al. [35] on subjects with acute coronary syndrome from Maracaibo. Furthermore, it should be noted that, in this study, the average age in females was 41 years, when a progressive decline in ovarian function tends to begin in most women [36]. In addition, females older than this average age displayed greater prevalence of DM2 and physical inactivity than their male counterparts; these factors favor endocrine-metabolic aging [37] which may explain our findings.

Indeed, aging has been closely linked with IR, chiefly due to the loss of lean mass and gain of visceral adiposity seen in this process [38]. Nonetheless, in multivariate analysis, we found individuals aged ≥60 years to have lower odds of IR. In this regard, certain reports have outlined a protective or innocuous role for adiposity in old age, due to reduced lipolytic activity in this tissue [39]. Likewise, in subjects with chronic-degenerative diseases, a BMI ≥ 25 Kg/m^2^ has been reported to be linked with lower mortality, in a phenomenon termed the “obesity paradox” [40, 41]. This effect may be responsible for the lower prevalence of IR in our study, as subjects aged ≥60 years exhibited high prevalence of abdominal obesity and comorbidities such as DM2, metabolic syndrome, and hypertension. At any rate, this relationship is very complex with numerous confounding factors, for example, smoking habits, and other comorbidities; therefore, future studies should further explore the interplay between obesity, IR, and old age.

When examining sociodemographic factors, we found marital status to be associated with IR, which was more frequent among married subjects. Notably, a large part of these were male, were inactive, and had elevated BMI and WC, possibly underlying this epidemiologic behavior. This harmonizes with the research by Sobal et al. [42] and Kiadaliri et al. [43] who described married men to have increased risk of overweight and obesity, associated with more frequent and abundant eating and decreased PA. Occupational status also displayed an interesting relationship with IR in our study, with employed individuals having increased odds for this condition. In this context, unemployed subjects exhibited higher leisure-domain PA, along with lower prevalence of elevated WC than their counterparts, in consonance with findings in an Iranian population where unemployment was a protective factor against overweight and obesity [43].

On the other hand, performance of any degree of leisure-domain PA, even under the recommended threshold, has been described to reduce global, cardiovascular, and cancer-related mortality [44], accompanied by a favorable impact on insulin sensitivity and glucose tolerance [45]. We found leisure-domain PA to be a protective factor against IR in males with >297 MET/week and females with >231 MET/week; thus, the American Diabetes Association recommendations on PA intensity and quantity may be applicable for prevention of DM2 in Maracaibo [46].

Alcohol consumption was found to be another risk factor for IR in our population. In a previous publication [14], we reported 45% of men and 16.7% of women in Maracaibo to be habitual drinkers; of these, 72.4% and 76.2% were shown to drink approximately 1–4 beers daily with a high prevalence of abdominal obesity in this group. In this respect, a meta-analysis by Bendsen et al. [47] encompassing 35 observational studies and 12 experimental studies determined high beer intake (>500 mL/day) to be associated with abdominal obesity, which in turn plays a central role in the development of IR.

We also found each of the separate components of the metabolic syndrome to be independently associated with IR, with the exception of hypertension. The relationship with obesity appears most prominent: In our population, BMI ≥ 30 kg/m^2^ grants 4 times greater odds of IR; this link has been extensively demonstrated in several cross-sectional and longitudinal studies [48, 49]. Similarly, high TAG has been associated with decreased insulin sensitivity [50], and both this disorder and low HDL-C have been independently linked with IR [51]. However, in our population, the mild association between elevated WC and IR appears to be dependent on the presence of DM2.

As may be expected, we also found high odds of IR in subjects with hyperglycemia; in this state, oxidation of fatty acids is attenuated, favoring their esterification and leading to a subsequent increase in synthesis of adipokines, which disrupt insulin activity and peripheral utilization of glucose [52]. In parallel, hyperglycemia potentiates oxidative stress via activation of the ceramide and diacylglycerol pathways and other intermediaries, which also hinder insulin signaling [53].

Our results show a close relationship between IR and various sociodemographic, psychobiologic, and cardiometabolic factors in our population, highlighting the clinician's role in the early detection of this condition. Indeed, regardless of the methods employed [54], identification of subjects at high cardiometabolic risk is a fundamental preventive strategy for reducing the morbidity and mortality of disorders associated with IR.

## Figures and Tables

**Table 1:** Gender-specific MET quintiles for each domain of physical activity (Maracaibo, Venezuela, 2015).

MET quintiles^*∗*^	Females		Males	
Work domain	Lower limit	Upper limit	Lower limit	Upper limit

Very low	33.00	385.99	33.00	714.99
Low	386.00	1201.49	715.00	2042.09
Moderate	1201.50	2751.59	2042.10	3578.39
High	2751.60	4546.79	3578.40	6495.59
Very high	4546.80		4546.80	

Transport domain	Lower limit	Upper limit	Lower limit	Upper limit

Very low	33.00	131.99	33.00	164.99
Low	132.00	230.99	165.00	257.49
Moderate	231.50	346.49	247.50	521.09
High	346.50	700.79	521.10	1385.99
Very high	700.80		1386.00	

Household domain	Lower limit	Upper limit	Lower limit	Upper limit

Very low	30.00	539.99	30.00	269.99
Low	540.00	1139.99	270.00	629.99
Moderate	1140.00	1919.99	630.00	1084.99
High	1920.00	3779.99	1085.00	2429.99
Very high	3780.00		2430.00	

Leisure domain	Lower limit	Upper limit	Lower limit	Upper limit

Very low	33.00	230.99	33.00	296.99
Low	321.00	445.49	297.00	791.99
Moderate	445.50	742.49	792.00	1532.39
High	742.50	1798.79	1532.40	2879.99
Very high	1798.80		2880.00	

^*∗*^Obtained from IPAQ scoring. Subjects with 0 MET were excluded from quintiles and classified separately as inactive.

**Table 2:** Sociodemographic and psychobiologic characteristics of the population by gender (Maracaibo, Venezuela, 2015).

	Females (*n* = 1056)		Males (*n* = 970)		Total (*n* = 2026)	
	*n*	%	*n*	%	*n*	%
*Age groups (years)*						
<30	308	29.2	365	37.6	673	33.2
30–59	607	57.5	517	53.3	1124	55.5
≥60	141	13.4	88	9.1	229	11.3

*Ethnic groups*						
Mixed	796	75.4	754	77.7	1550	76.5
Hispanic white	175	16.6	146	15.1	321	15.8
Afro-Venezuelan	27	2.6	33	3.4	60	3.0
American Indian	47	4.5	36	3.7	83	4.1
Others	11	1.0	1	0.1	12	0.6

*Socioeconomic status*						
Stratum I	17	1.6	19	2.0	36	1.8
Stratum II	179	17.0	185	19.1	364	18.0
Stratum III	394	37.3	409	42.2	803	39.6
Stratum IV	411	38.9	325	33.5	736	36.3
Stratum V	55	5.2	32	3.3	87	4.3

*Marital status*						
Single	445	42.3	414	43.1	859	42.7
Married	392	37.2	417	43.4	809	40.2
Others	216	20.5	130	13.5	346	17.2

*Educational status*						
Illiterate	25	2.4	17	1.8	42	2.1
Primary education	217	20.5	103	10.6	320	15.8
Secondary education	466	44.1	479	49.4	945	46.6
High education	348	33.0	371	38.2	719	35.5

*Occupational status*						
Unemployed	575	54.5	263	27.1	838	41.4
Employed	481	45.5	707	72.9	1188	58.6

*Smoking habit*						
Nonsmoker	792	75.1	606	62.9	1398	69.3
Current smoker	119	11.3	181	18.8	300	14.9
Past smoker	143	13.6	177	18.4	320	15.9

*Alcohol intake* ^*∗*^ * (%)*	196	18.6	470	48.5	666	32.9

*Physical inactivity* ^*∗∗*^ * (%)*	252	23.9	443	45.7	695	34.3

^*∗*^≥1 g of alcohol per day. ^*∗∗*^Based on IPAQ scoring.

**Table 3:** Anthropometric and cardiometabolic characteristics of the population by gender (Maracaibo, Venezuela, 2015).

	Females (*n* = 1056)		Males (*n* = 970)		Total (*n* = 2026)	
	*n*	%	*n*	%	*n*	%
*Body Mass Index (kg/m* ^*2*^)						
≤24.9	377	35.7	245	25.3	622	30.7
25–29.9	333	31.5	386	39.8	719	35.5
≥30	346	32.8	339	34.9	685	33.8

*High TAG* ^‡^						
Absent	815	77.2	644	66.4	1459	72.0
Present	241	22.8	326	33.6	567	28.0

*Low HDL-C* ^‡^						
Absent	384	36.4	479	49.4	863	42.6
Present	672	63.6	491	50.6	1163	57.4

*Elevated WC* ^*∗*^						
Absent	597	51.0	502	47.4	1099	49.3
Present	573	49.0	556	52.6	1129	50.7

*JNC-8 classification*						
Normotensive	481	45.5	312	32.2	793	39.1
Prehypertensive	381	36.1	384	39.6	765	37.8
Hypertensive	194	18.4	274	28.2	468	23.1

*Glycemic status* ^*¥*^						
Normoglycemic	775	73.5	667	68.8	1442	71.2
Impaired fasting glycemia	187	17.7	217	22.4	404	20.0
Type 2 diabetes mellitus	92	8.7	86	8.9	178	8.8

TAG: triacylglycerides; HDL-C: high-density lipoprotein-cholesterol; WC: waist circumference.

^‡^ *IDF/AHA/NHLBI/WHF/IAS/IASO* 2009 consensus criteria.

^*∗*^Females ≥ 90 cm; males ≥ 95 cm.

^¥^ *ADA* 2014 classification.

**Table 4:** Prevalence of insulin resistance in the general population by sociodemographic variables (Maracaibo, Venezuela, 2015).

	HOMA2-IR < 2		HOMA2-IR ≥ 2		*p* ^*∗*^	*χ* ^2^(*p*)
	*n*	%	*n*	%		
*Ethnic group*						*2.65 (0.61)*
Mixed	837	54.0	713	46.0	NS	
Hispanic white	161	50.2	160	49.8	NS	
Afro-Venezuelan	33	55.0	27	45.0	NS	
American Indian	47	56.6	36	43.4	NS	
Others	5	41.7	7	58.3	NS	

*Socioeconomic status*						*4.33 (0.36)*
Stratum I: High Class	18	50.0	18	50.0	NS	
Stratum II: Middle-High Class	186	51.1	178	48.9	NS	
Stratum III: Middle Class	445	55.4	358	44.6	NS	
Stratum IV: Relative Poverty	382	51.9	354	48.1	NS	
Stratum V: Critical Poverty	52	59.8	35	40.2	NS	

*Marital status*						*17.29 (0.001)*
Single	503	58.6	356	41.4	<0.05	
Married	392	48.5	417	51.5	<0.05	
Others	181	52.3	165	47.7	NS	

*Educational status*						*6.76 (0.079)*
Illiterate	27	64.3	15	35.7	NS	
Primary education	154	48.1	166	51.9	<0.05	
Secondary education	521	55.1	424	44.9	NS	
High education	381	53.0	338	47.0	NS	

*Occupational status*						*3.25 (0.071)*
Unemployed	428	51.1	410	48,9	NS	
Employed	655	55.1	533	44.9	NS	

NS: nonsignificant; *p* ^*∗*^: *Z* test for proportions; *χ* ^2^: Pearson's Chi-squared test.

**Table 5:** Prevalence of insulin resistance in the general population by physical activity domains (Maracaibo, Venezuela, 2015).

Physical activity pattern	HOMA2-IR < 2		HOMA2-IR ≥ 2		*p* ^*∗*^	*χ* ^2^(*p*)
	*n*	%	*n*	%		
*Work domain*						*1.82 (0.87)*
Inactive	799	53.1	706	46.9	NS	
Very low	56	53.8	48	46.2	NS	
Low	63	58.9	44	41.1	NS	
Moderate	52	51.0	50	49.0	NS	
High	54	52.9	48	47.1	NS	
Very high	59	55.7	47	44.3	NS	

*Transport domain*						*8.60 (0.12)*
Inactive	359	49.3	369	50.7	NS	
Very low	129	56.3	100	43.7	NS	
Low	147	56.1	115	43.9	NS	
Moderate	142	55.5	114	44.5	NS	
High	161	57.1	121	42.9	NS	
Very high	132	54.8	109	45.2	NS	

*Household domain*						*2.27 (0.81)*
Inactive	295	52.2	270	47.8	NS	
Very low	151	55.7	120	44.3	NS	
Low	160	53.5	139	46.5	NS	
Moderate	172	56.2	134	43.8	NS	
High	148	52.5	134	47.5	NS	
Very high	157	51.8	146	48.2	NS	

*Leisure domain*						*23.24 (0.003)*
Inactive	612	50.0	613	50.0	NS	
Very low	79	50.3	78	49.7	NS	
Low	98	57.6	72	42.4	NS	
Moderate	92	59.0	64	41.0	NS	
High	92	61.3	58	38.7	<0.05	
Very high	110	65.5	58	34.5	<0.05	

NS: nonsignificant; *χ* ^2^: Pearson's Chi-squared test; *p* ^*∗*^: *Z* test for proportions.

**Table 6:** Prevalence of insulin resistance in the general population by metabolic characteristics (Maracaibo, Venezuela, 2015).

	HOMA2-IR < 2		HOMA2-IR ≥ 2		*p* ^*∗*^	*χ* ^2^(*p*)
	*n*	%	*n*	%		
*Body Mass Index (kg/m* ^*2*^)						*232.022 (<0.001)*
≤24.9	456	73.3	166	26.7	<0.05	
25–29.9	409	57.4	305	42.6	<0.05	
≥30	214	31.2	469	68.8	<0.05	

*High TAG* ^‡^						*83.47 (5.42 × 10* ^−*20*^)
Absent	872	59.8	587	40.2	<0.05	
Present	211	37.2	356	62.8	<0.05	

*Low HDL-C* ^‡^						*38.27 (6.15 × 10* ^−*10*^)
Absent	530	61.4	333	38.6	<0.05	
Present	553	47.5	610	52.5	<0.05	

*Elevated WC* ^*∗*^						*198.75 (<0.001)*
Absent	689	63.7	304	32.3	<0.05	
Present	393	36.3	638	67.7	<0.05	

*JNC-8 classification*						*42.83 (<0.001)*
Normotensive	484	61	309	39	<0.05	
Prehypertensive	402	52.5	363	47.5	NS	
Hypertensive	197	42.1	271	57.9	<0.05	

*Glycemic status* ^*¥*^						*155.37 (<0.001)*
Normoglycemic	892	61.9	550	38.1	<0.05	
Impaired fasting glycemia	152	37.6	252	62.4	<0.05	
Type 2 diabetes mellitus	38	21.3	140	78.7	<0.05	

^*∗*^ *Z* test for proportions; *χ* ^2^: Pearson's Chi-squared test.

TAG: triacylglycerides; HDL-C: high-density lipoprotein-cholesterol; WC: waist circumference.

^‡^ *IDF/AHA/NHLBI/WHF/IAS/IASO *2009 consensus criteria.

^*∗*^Females ≥ 90 cm; males ≥ 95 cm.

^*¥*^ *ADA* 2014 classification.

**Table 7:** Biologic and anthropometric characteristics in the general population by insulin resistance status (Maracaibo, Venezuela, 2015).

	HOMA2-IR < 2 (*n* = 1083)	HOMA2-IR ≥ 2 (*n* = 943)	*p* ^*∗*^
Age (years)^a^	38.48 ± 15.50	41.09 ± 15.11	3.15 × 10^−5^
Body Mass Index (kg/m^2^)^a^	26.39 ± 5.24	30.60 ± 6.52	7.13 × 10^−55^
Waist circumference (cm)^a^	89.92 ± 12.68	100.34 ± 16.6	4.49 × 10^−54^
Fasting glycemia (mg/dL)^a^	91.88 ± 20.99	107.48 ± 50.78	3.41 × 10^−39^
Fasting insulin (*µ*U/mL)^a^	8.80 ± 2.70	21.46 ± 10.15	<0.001
HOMA2-IR^a^	1.31 ± 0.40	3.24 ± 1.47	<0.001
Total cholesterol (mg/dL)^a^	186.91 ± 45.67	196.87 ± 46.31	2.39 × 10^−7^
Triacylglycerides (mg/dL)^a^	110.70 ± 76.27	154.48 ± 122.02	5.45 × 10^−29^
LDL-C (mg/dL)^a^	118.80 ± 38.06	125.25 ± 38.84	<0.001
HDL-C (mg/dL)^a^	45.90 ± 12.58	41.97 ± 10.95	3.67 × 10^−14^
Systolic BP (mmHg)^a^	117.91 ± 16.57	121.96 ± 17.08	2.33 × 10^−8^
Diastolic BP (mmHg)^a^	75.75 ± 10.99	79.05 ± 11.44	2.93 × 10^−11^
hs-CRP (mg/dL)^b^	0.313 (0.086–0.622)	0.420 (0.154–0.942)	4.51 × 10^−7^

LDL-C: low-density lipoprotein-cholesterol; HDL-C: high-density lipoprotein-cholesterol; BP: blood pressure; hs-CRP: High-Sensitivity C-Reactive Protein.

^a^Normally distributed variable, expressed as mean ± standard deviation; ^b^nonnormally distributed variable, expressed as median (25th percentile–75th percentile).

^*∗*^Student's *t*-test was used for normally distributed variables; Mann-Whitney *U* test was used for nonnormally distributed variables.

**Table 8:** Sociodemographic and metabolic risk factors associated with insulin resistance (Maracaibo, Venezuela, 2015).

			*Model 1* ^*∗*^		*Model 2* ^*∗*^	
	Crude odds ratio (CI 95%^a^)	*p* ^b^	Adjusted odds ratio (CI 95%^a^)	*p* ^b^	Adjusted odds ratio (CI 95%^a^)	*p* ^b^
*Gender*						
Males	1.00	—	1.00	—	1.00	—
Females	1.01 (0.85–1.21)	0.89	1.29 (1.02–1.65)	0.04	1.33 (1.04–1.71)	0.02

*Age groups (years)*						
<30	1.00	—	1.00	—	1.00	—
30–59	1.48 (1.22–1.80)	<0.01	0.65 (0.50–0.85)	<0.01	0.64 (0.49–0.84)	<0.01
≥60	1.50 (1.11–2.02)	<0.01	0.42 (0.28–0.65)	<0.01	0.42 (0.27–0.66)	<0.01

*Occupational status*						
Unemployed	1.00	—	1.00	—	1.00	—
Employed	1.31 (1.09–1.58)	<0.01	1.45 (1.15–1.83)	<0.01	1.42 (1.12–1.80)	<0.01

*Leisure-domain physical activity*						
Inactive	1.00	—	1.00	—	1.00	—
Very low	0.99 (0.71–1.37)	0.93	1.25 (0.85–1.86)	0.24	1.29 (0.87–1.93)	0.21
Low	0.73 (0.53–1.01)	0.06	0.71 (0.48–1.04)	0.07	0.70 (0.47–1.04)	0.08
Moderate	0.69 (0.49–0.97)	0.04	0.76 (0.51–1.14)	0.17	0.72 (0.48–1.09)	0.12
High	0.63 (0.45–0.89)	<0.01	0.69 (0.46–1.03)	0.07	0.63 (0.41–0.95)	0.03
Very high	0.53 (0.38–0.74)	<0.01	0.77 (0.52–1.15)	0.19	0.80 (0.54–1.20)	0.28

*Alcohol intake*						
Absent	1.00	—	1.00	—	1.00	—
Present	1.21 (1.00–1.47)	0.05	1.41 (1.10–1.79)	<0.01	1.44 (1.12–1.84)	<0.01

*High TAG* ^‡^						
Absent	1.00	—	1.00	—	1.00	—
Present	2.51 (2.05–3.06)	<0.01	1.77 (1.38–2.27)	<0.01	1.76 (1.36–2.29)	<0.01

*Low HDL-C* ^‡^						
Absent	1.00	—	1.00	—	1.00	—
Present	1.76 (1.47–2.10)	<0.01	1.31 (1.06–1.64)	0.01	1.33 (1.06–1.66)	0.01

*Elevated waist circumference*						
Absent	1.00	—	1.00	—	1.00	—
Present	3.68 (3.06–4.42)	<0.01	1.31 (0.98–1.76)	0.07	1.28 (0.95–1.73)	0.11

*JNC-8 classification*						
Normotensive	1.00	—	1.00	—	1.00	—
Prehypertensive	1.41 (1.16–1.73)	<0.01	0.96 (0.75–1.22)	0.73	0.96 (0.75–1.23)	0.75
Hypertensive	2.16 (1.71–2.72)	<0.01	0.99 (0.73–1.37)	0.99	1.06 (0.77–1.46)	0.73

*Body Mass Index (kg/m* ^*2*^)						
≤24.9	1.00	—	1.00	—	1.00	—
25–29.9	2.04 (1.62–2.57)	<0.01	1.90 (1.41–2.55)	<0.01	1.93 (1.43–2.61)	<0.01
≥30	6.05 (4.76–7.69)	<0.01	4.33 (2.97–6.29)	<0.01	4.40 (2.99–6.47)	<0.01

*Glycemic status* ^*¥*^						
Normoglycemic	1.00	—	1.00	—	1.00	—
Impaired fasting glycemia	2.69 (2.14–3.38)	<0.01	2.43 (1.87–3.15)	<0.01	2.44 (1.88–3.17)	<0.01
Type 2 diabetes mellitus	5.98 (4.11–8.68)	<0.01	4.62 (2.71–7.88)	<0.01	—	—

^a^Confidence interval (95%); ^b^significance.

Model 1: adjusted for gender, age groups, ethnic groups, socioeconomic status, educational status, marital status, occupational status, diabetes mellitus family history, alcohol intake, smoking habits, leisure-domain physical activity, glycemic status, presence of high TAG, low HDL-C, and elevated waist circumference, BMI classification and JNC-8 classification.

Model 2: excluding diabetic subjects.

^*¥*^ *ADA* 2014 classification.

^‡^ *IDF/AHA/NHLBI/WHF/IAS/IASO *2009 consensus criteria.
